# The Effect of Sleep Quality on Coronary Lesion Severity and Prognosis in the Young Acute Coronary Syndrome Population

**DOI:** 10.3390/jcdd11020068

**Published:** 2024-02-19

**Authors:** Jiaxin Yang, Kexin Wang, Wenjie Wang, Jialong Niu, Xiaoli Liu, Hua Shen, Yan Sun, Hailong Ge, Hongya Han

**Affiliations:** 1 Department of Cardiology, Beijing Anzhen Hospital, Capital Medical University, Beijing 100029, China; yangjiaxin1213@163.com (J.Y.); wangkx_1998@163.com (K.W.); 13120043661@163.com (W.W.); njl113735@163.com (J.N.); liuxiaoli@ccmu.edu.cn (X.L.); shenhua@ccmu.edu.cn (H.S.); sunnyan0616@126.com (Y.S.); 2 Department of Cardiology, Beijing Hospital, National Center of Gerontology, Institute of Geriatric Medicine, Chinese Academy of Medical Sciences, Peking Union Medical College, Beijing 100730, China; 3 Department of Cardiology, Beijing Geriatric Hospital, Beijing 100095, China

**Keywords:** sleep quality, acute coronary syndrome, cardiovascular events, SYNTAX score, Pittsburgh sleep quality index

## Abstract

This study aimed to explore the effect of long-term (≥1 year) sleep quality on coronary lesion complexity and cardiovascular prognosis in young acute coronary syndrome (ACS) patients. We consecutively recruited young patients aged from 18 to 44 years old with first-episode ACS and significant epicardial stenosis on coronary angiography from January 2016 to January 2017. Coronary lesion complexity was evaluated based on SYNTAX scores. Long-term sleep quality was assessed using the Pittsburgh Sleep Quality Index (PSQI) (PSQI ≤ 5 and PSQI > 5 groups). The primary endpoints were major adverse cardiovascular events (MACEs). A total of 466 young ACS patients (93.13% male; median age, 41 years) were included. Poor sleepers (PSQI > 5) had higher SYNTAX scores. After adjusting for confounders, PSQI scores (continuous variables, OR: 1.264; 95%CI: 1.166–1.371; *p* < 0.001) and PSQI grade (binary variable, OR: 3.864; 95%CI: 2.313–6.394; *p* = 0.001) were significantly associated with an increased risk of complex coronary lesions. During a median follow-up of 74 months, long-term poor sleep quality (PSQI > 5) was significantly associated with an increased risk of MACEs (HR: 4.266; 95%CI: 2.274–8.001; *p* < 0.001). Long-term poor sleep quality was a risk factor for complex coronary lesions and has adverse effects on cardiovascular prognosis in the young ACS population.

## 1. Introduction

With economic and social development and changes in people’s lifestyles, the incidence of acute coronary syndrome (ACS) among young adults is increasing year by year [1]. The World Health Organization uses a special classification to define coronary artery disease (CAD) occurring before the age of 45 years as youth CAD. It has different characteristics than elderly coronary heart disease; the lesions occur earlier, and the disease progresses more rapidly [2]. For young patients, increased risks of major adverse cardiovascular events (MACEs) have been observed after ACS. Thus, it is essential to identify the editable risk factors of ACS and to carry out early intervention to delay its incidence. The development of ACS is always associated with inflammation [3,4]. Thus, it has been demonstrated that male sex, hypertension, diabetes, hyperlipidemia, obesity, smoking, and insufficient sleep are risk factors for cardiovascular disease mortality in the young population [5,6,7].

As an important neurobehavioral state, sleep has been an essential topic in abundant genetic, epigenetic, and proteomic studies. Robust evidence has revealed that poor sleep quality may cause adverse immunological and metabolic changes, including increased susceptibility to infectious disease, decreases in growth hormone, and increases in sympathetic activation [8]. Moreover, poor sleep quality could also increase the risk of cognitive impairments and other chronic diseases, such as stroke, type 2 diabetes [9], calcification of the coronary arteries [10], and death [11,12]. Growing evidence indicates that sleep duration and sleep disorder are also associated with a higher risk of ACS and MACEs [13,14]. However, clinical evidence on the effect of sleep quality on coronary lesion complexity and cardiovascular prognosis in youth ACS is inadequate and warrants further study. Therefore, we conducted this prospective cohort study to investigate the effect of sleep quality on coronary lesion severity and cardiovascular prognosis in young ACS patients.

## 2. Materials and Methods

### 2.1. Study Procedures and Population

This cohort study consecutively enrolled young patients aged 18 to 44 years old with suspected ACS who were admitted to Beijing Anzhen Hospital from January 2016 to January 2017. Participants were excluded if they refused coronary angiography (CAG) or had a history of myocardial infarction (MI), percutaneous coronary intervention (PCI), coronary artery bypass graft surgery (CABG), severe heart failure (New York Heart Association classes III–IV or Killip classes III–IV), severe renal insufficiency (estimated glomerular filtration rate < 30 mL/min/1.73 m^2^), severe valvular heart disease, malignant arrhythmia (ventricular tachycardia, ventricular fibrillation, high atrioventricular block, etc.), congenital heart disease, myocarditis, cardiomyopathy, heart transplantation, secondary hypertension, or severe wasting disease (malignant tumor, liver disease, severe infection, etc.). We took complete medical histories and performed fasting blood tests and electrocardiograms (ECGs) on all included patients. In addition, sleep questionnaires and CAG were also completed. Based on the results of CAG, the cardiologist recommends an individualized treatment to the patient, including PCI, CABG, or conservative treatment. According to the guidelines [15] and combined with auxiliary examinations, patients who had significant epicardial stenosis after CAG and who were diagnosed with ACS were included in this study. For the enrolled patients, clinical follow-up was performed semiannually, ending in August 2022, to record data on their sleep changes, clinical status, all interventions received, and outcome events.

**Figure 1:**
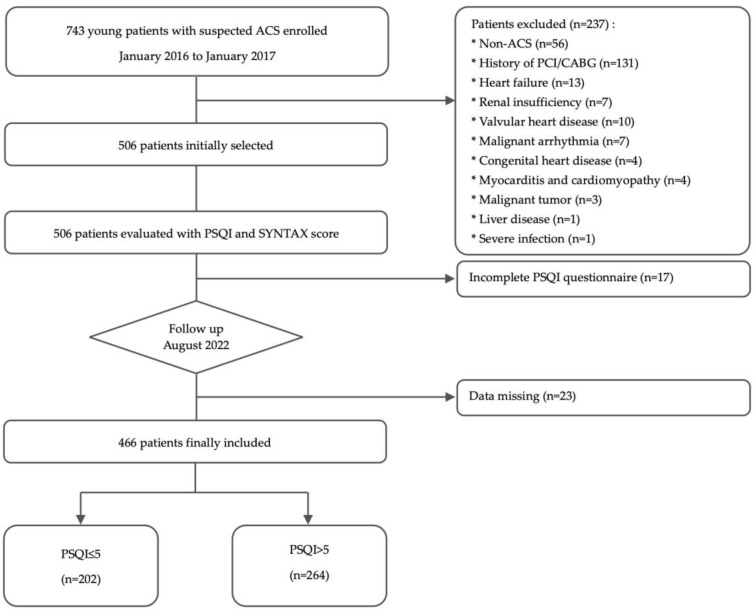
Flow chart of enrollment. ACS = acute coronary syndrome; PCI = percutaneous coronary intervention; CABG = coronary artery bypass graft surgery; PSQI = Pittsburgh Sleep Quality Index.

A total of 743 young patients with suspected ACS were consecutively enrolled between January 2016 and January 2017. After excluding 237 patients according to the criteria, the PSQI questionnaires were administered and SYNTAX scores were recorded in the remaining 506 patients. During the research process, 17 patients were removed because of incomplete questionnaire content, and 23 patients were lost during the follow-up. The missing outcomes were less than 5% of the total. Finally, 466 patients were included and divided into a PSQI ≤ 5 group (*n* = 202) and a PSQI > 5 group (*n* = 264) (Figure 1). The study was approved by the Ethics Committee of Beijing Anzhen Hospital, Capital Medical University (No. 2016044X). All enrolled patients provided written informed consent.

### 2.2. Diagnostic Criteria

Hypertension was diagnosed when it was consistent with 2 or more systolic blood pressures ≥ 140 mmHg or diastolic blood pressures ≥ 90 mmHg [16]. Diabetes mellitus was diagnosed when it was consistent with fasting glucose ≥ 7.0 mmol/L or non-fasting glucose ≥ 11.10 mmol/L [17], and hyperlipidemia was diagnosed when it was consistent with fasting total cholesterol > 5.18 mmol/L or triglyceride > 1.72 mmol/L [18]. Smoking included all former and current smokers. Patients were also classified as hypertensive, diabetic, or hyperlipidemic if they had been clearly diagnosed and/or were taking medication.

### 2.3. Coronary and Sleep Assessment

Coronary lesion complexity was assessed using the SYNTAX score (SS) [19], which was recorded by two experienced cardiologists based on CAG images with the assistance of a senior cardiovascular imaging specialist if there was a discrepancy between the scores. According to the guidelines [20], coronary lesion complexity was divided into a low-complexity group with SS ≤ 22 and a high-complexity group with SS > 22.

Sleep quality was assessed with the Pittsburgh Sleep Quality Index (PSQI) questionnaire based on the participants’ sleep habits over the past year before CAG. The investigators were highly trained and unaware of the patient’s diagnoses or coronary angiography findings. PSQI is one of the most widely used standardized measures to evaluate sleep quality and has shown good internal reliability and validity in a variety of samples [21,22]. This questionnaire can be used for different continents, countries, genders, and ages, and in patients with sleep disorders, psychiatric disorders, or cancer [23,24,25]. It is composed of 18 individual items, comprising 7 “component” scores: subjective sleep quality, sleep latency, sleep duration, habitual sleep efficiency, sleep disturbances, use of sleeping medication, and daytime dysfunction [26]. Each component has a score scale of 0 to 3 with ‘0’ indicating no difficulty and ‘3’ indicating great difficulty [26]. The seven components were added together to form a ‘PSQI score’ ranging from 0 to 21 with ‘0’ indicating no difficulty and ‘21’ indicating severe difficulty in all areas. Grades of PSQI ≤ 5 and PSQI > 5 are used to represent good and poor sleep quality according to the original paper [26], and this has been confirmed with known-group validity in many studies [24,27].

### 2.4. Study Endpoints

The primary endpoints were MACEs, which were defined as the composite of all-cause death, MI, and target-vessel revascularization (TVR). Secondary endpoints included all-cause death, cardiac death, MI, and TVR.

All deaths were considered to be due to cardiac causes unless a non-cardiac cause could be identified. MI was defined as symptoms, ECG changes, or abnormal imaging with a creatine kinase MB fraction above the upper normal limits or a troponin T/I level above the 99th percentile of the upper normal limit [28]. TVR was defined as a repeat PCI/CABG in the target vessel either with the presence of ischemic symptoms or a positive stress test and ≥50% stenosis of the vessel diameter or with the absence of ischemic symptoms or a negative stress test and ≥70% stenosis of the vessel diameter [29]. Cardiac death was defined as death due to MI, cardiac perforation, or pericardial tamponade; arrhythmia within 30 days of the procedure or related to the procedure; death due to a procedural complication; or any case of death in which a cardiac cause was not excluded by a clinical event committee [30]. If >1 event occurred during follow-up (death > MI > TVR), the most severe endpoint event was selected for the primary endpoint analysis.

### 2.5. Statistical Analyses

Normally distributed continuous variables were expressed as mean ± standard deviation, and non-normally distributed variables were expressed as the medians and interquartile range (IQR) (Q25–Q75). Categorical variables are presented as numbers (percentages). Differences in continuous variables were analyzed with Student’s *t*-test or the Mann–Whitney U test, and differences in categorical variables were analyzed with the chi-square test or Fisher’s exact test, as appropriate. Multifactorial logistic regression was used to analyze the association between sleep quality, as a dichotomous variable (“PSQI grade”) and a continuous variable (“PSQI score”), and coronary lesion complexity. In addition, the relationship between time to sleep and sleep duration, as independent variables, and SYNTAX scores were listed separately. On this basis, subgroup analyses were performed to determine the interaction between sleep quality and other risk factors. Cox proportional hazard regression was used to assess the association between PSQI grade (dichotomous variable) and MACEs, while the Kaplan–Meier method (log-rank test) was used to determine the cumulative incidence of MACEs. Clinically significant confounders and variables with statistical significance (*p* < 0.15) from univariate analysis were included in the model after being checked for collinearity. All statistical analyses were performed with R version 4.1.3 (R Foundation for Statistical Computing, Vienna, Austria) and IBM SPSS Statistics version 26.0 (IBM Corporation, Chicago, IL, USA). Two-tailed *p*-values of < 0.05 were considered statistically significant.

## 3. Results

### 3.1. Baseline Characteristics

In total, 93.13% (434) of the patients were male; the median age and body mass index (BMI) were 41 (37–43) years and 27.4 (25.28–29.4) kg/m^2^, respectively, and 56.44% (263), 30.04% (140), and 88.63% (413) had hypertension, diabetes, and hyperlipidemia, respectively. Smokers accounted for 62.45% (291). In addition, for coronary lesion complexity, the median SYNTAX score was 14 (7–23), of which 25.32% (118) had high complexity (SS > 22).

Compared with patients with good sleep quality (PSQI ≤ 5), patients with poor sleep quality (PSQI > 5) had higher SYNTAX scores (*p* < 0.001). The proportion of stenosis was higher in the left main (LM) and left circumflex (LCX) (all *p* < 0.05). We also found that patients with poor sleep quality had higher hypersensitive c-reactive protein (hs-CRP) (*p* = 0.049) and were more likely to use aspirin (*p* = 0.013) and statins (*p* = 0.031). There were no significant differences in other clinical, laboratory, or medication history characteristics between the two groups (Table 1 and Table 2).

### 3.2. Association of Sleep Quality and Coronary Lesion Complexity

Univariate logistic regression analysis (Supplementary Table S1) showed that with the SYNTAX score as the dependent variable, diabetes (OR: 1.726; 95%CI: 1.113–2.678; *p* = 0.015), fasting blood glucose (OR: 1.067; 95%CI: 1.004–1.135; *p* = 0.038), high-density lipoprotein cholesterol (OR: 0.241; 95%CI: 0.077–0.755; *p* = 0.015), time for bed (OR: 1.413; 95%CI: 1.136–1.758; *p* = 0.002), PSQI score (OR: 1.265; 95%CI: 1.171–1.368; *p* < 0.001), and PSQI grade (OR: 3.851; 95%CI: 2.361–6.279; *p* < 0.001) were all associated with more complex coronary lesions, and sleep duration was a protective factor (OR: 0.764; 95%CI: 0.630–0.927; *p* = 0.006). After adjusting for clinically significant confounders in the analysis (Model 1 and Model 2), the results were similar to those above.

After incorporating clinically significant confounding factors and variables that were statistically significant (*p* < 0.15) in the univariate analysis into Model 3, time for bed (OR: 1.457; 95%CI: 1.156–1.836; *p* = 0.001), PSQI score (OR: 1.264; 95%CI: 1.166–1.371; *p* < 0.001), and PSQI grade (OR: 3.864; 95%CI: 2.313–6.394; *p* = 0.001) remained significantly associated with an increased risk of high coronary lesion complexity, but long sleep duration (OR: 0.775; 95%CI: 0.633–0.948; *p* = 0.013) might reduce this risk (Table 3). A collinearity check of covariates is shown in Supplementary Table S2.

**Figure 2:**
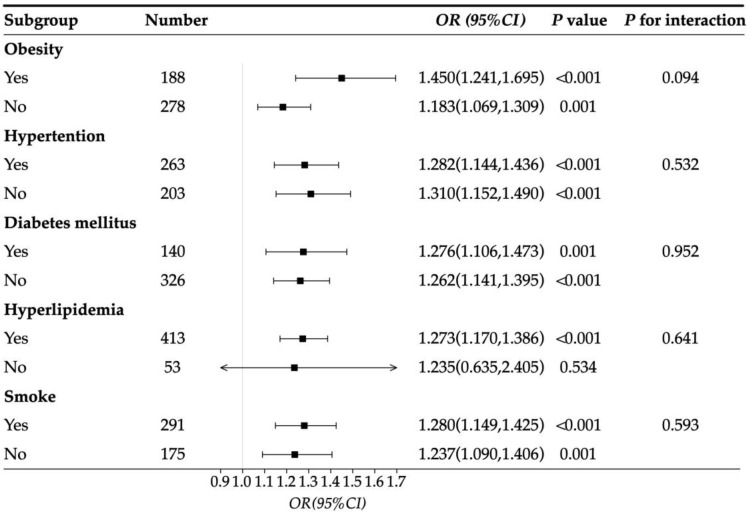
The subgroup analyses for the relationship between PSQI score and other risk factors. Adjusted for sex, age, BMI, hypertension, diabetes mellitus, hyperlipidemia, smoker, FBG, HbA1c, HDL-C, Uric acid, and Hcy.

We also performed subgroup analyses based on Model 3 to investigate whether there was any interaction between PSQI scores and other risk factors, such as obesity, hypertension, diabetes mellitus, hyperlipidemia, and smoking, but the results were negative (*p* for interaction > 0.05) (Figure 2).

### 3.3. Association of Sleep Quality and Outcomes

**Figure 3:**
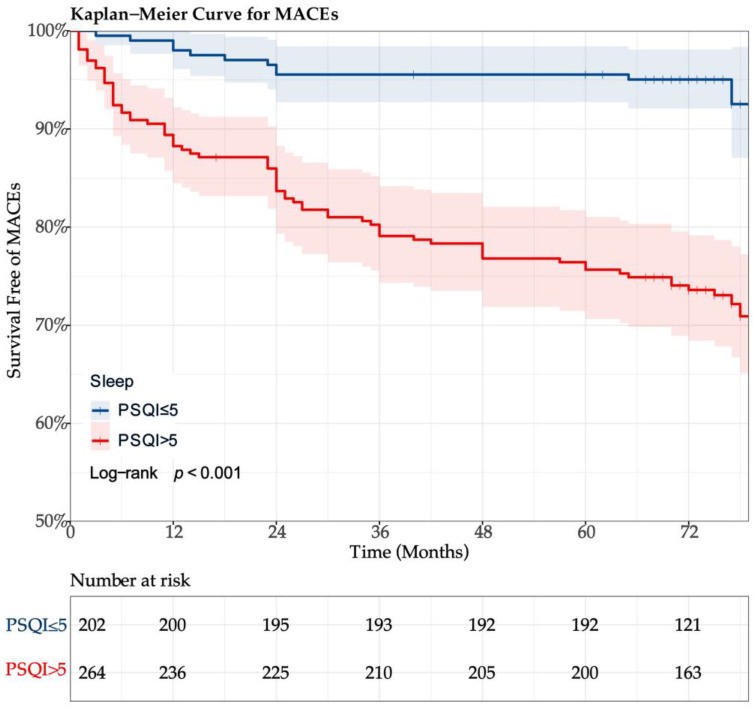
Kaplan–Meier curves of survival free of MACEs and PSQI grades.

During a median follow-up period of 74 (69–77) months, MACEs occurred in 84 (18.03%) of 466 participants: 4 (0.86%) cases of all-cause death, 17 (3.65%) cases of MI, and 82 (17.60%) cases of TVR. Of those who died from all causes, 2 (0.43%) were cardiac deaths. In Cox proportional hazard regression analysis, endpoint events were used as the dependent variable, and the PSQI grade (categorical variable) was used as the independent variable. Clinically significant confounding factors and variables with statistical significance (*p* < 0.15) in the univariate analysis (Supplementary Table S3) were included in the regression model. Compared with patients in the PSQI ≤ 5 group, MACEs (HR: 4.266; 95%CI: 2.274–8.001; *p* < 0.001) and TVR (HR: 4.073; 95%CI: 2.169–7.652; *p* < 0.001) significantly increased (Table 4). Although the incidence of myocardial infarction in PSQI > 5 was 20.8% higher than in PSQI ≤ 5, the difference was not statistically significant (HR: 1.208; 95%CI: 0.392–3.725; *p* = 0.317). The Kaplan–Meier curve (Figure 3) showed that PSQI > 5 was significantly associated with an increased cumulative risk of MACEs (log-rank test, *p* < 0.001). A collinearity check of covariates is shown in Supplementary Table S4.

## 4. Discussion

To the best of our knowledge, the young population represents a special cohort that deserves more attention regarding the prevention of ACS and its malignant outcomes. This prospective study with a 5-year follow-up investigated the role of poor sleep quality on coronary lesion complexity and MACEs in young ACS patients and elicited three main findings. First, high PSQI scores, which usually represent poor sleep quality, were positively correlated with coronary lesion complexity. Second, after adjustment for confounding factors, long-term poor sleep quality (PSQI > 5) was found to be an independent risk factor for MACEs, especially TVR. In addition, our subgroup analysis showed no significant interaction relationships between PSQI scores and hypertension, diabetes mellitus, hyperlipidemia, or smoking status in the process of ACS. Taken together, this is the first study to show that a high PSQI score is a predictor of coronary lesion complexity and is significantly associated with the occurrence of MACEs in young ACS patients. This might provide new information about the prevention and rehabilitation of ACS.

In recent years, the incidence of ACS among the young population (under the age of 45) has significantly increased [1,31]. Compared with those in the elderly, the risk factors of ACS in young people have different characteristics, which are closely related to abnormal living habits such as smoking, less physical activity, excessive stress, and sleep disorders [32,33]. As our literature review illustrated, short sleep duration, shift work, and poor sleep quality could all promote adverse health, including headache, stroke, gastrointestinal disorders, and cardiovascular disease [34,35,36,37]. Zhao et al. demonstrated that shift work can increase myocardial infarction reperfusion injuries and the incidence of heart failure in mouse, ovine, and human models after acute MI, which might be associated with an elevated risk of MACEs [13]. Post-ACS patients with a history of overnight shift work were found to be at increased risk of MACEs in a long-term follow-up period in [38]. Moreover, in the Sleep Heart Health Study, short sleep duration (<7.5 h per night) was predictive of incident stroke or MI (with HR = 1.68) [39]. Compared with normal sleep duration, all-cause death has been illustrated to be higher in short-duration (≤5 h per night) sleep and prolonged-duration (>9 h) sleep in post-ACS patients [40]. In addition, Barger et al. demonstrated that obstructive sleep apnea (OSA) is underrecognized as a predictor of adverse outcomes after ACS [14]. Furthermore, a systematic review and meta-analysis involving 122,501 subjects found that the sleep disorder insomnia could also lead to an increased risk of developing MACEs or death from cardiovascular disease during the follow-up period (RR 1.45, 95%CI 1.29–1.62, with *p* < 0.01) [41]. However, although abundant research has discussed how shift work, sleep duration, and disorders affect cardiovascular health, few studies have focused on poor sleep quality using specific scores.

In the present study, we used a variety of approaches to analyze data from a youth population cohort. Thus, we are the first to unveil the association between sleep quality and cardiovascular prognosis in young ACS patients. We demonstrated that poor sleep quality is positively correlated with high coronary lesion complexity and adverse prognosis in young ACS patients in a long-term follow-up period. Unlike the studies mentioned above, our study focused on poor sleep quality evaluated with the PSQI. High PSQI scores (usually > 5) have been considered to show poor subjective sleep quality in previous research [25]. After using different models of multivariate logistic regression analysis, the results showed that there was a significant association between PSQI scores and elevated SYNTAX scores, which also represented the complexity of coronary lesions [42]. Thus, we concluded that long-term poor sleep quality is an independent risk factor for high-complexity coronary lesions in youth ACS, which is consistent with the results of previous studies. Another important finding of the current study is that poor long-term sleep quality is one of the factors that influences poor prognosis in young ACS patients. Based on a five-year follow-up, we demonstrated that patients with higher PSQI scores were more likely to suffer from TVR. The results were clearly driven by TVR, so although sleep quality is related to a higher SYNTAX score, it is likely that worse outcomes are related to more complex coronary disease. Of note, there was no significant difference in the rate of MI, all-cause death, or cardiac death because of the limited number of positive cases. Further research needs to be carried out to identify young ACS patients at high risk of MACEs. In addition, unlike previous studies, we did not find an association between hypertension, diabetes, hyperlipidemia, smoking, and sleep quality in subgroup analyses, which may be related to our small sample size. In our study, 93.13% of young ACS patients were male. The reason may be that male patients have more high-risk factors such as smoking, alcohol abuse, obesity, and high-fat diets. In addition, young women are protected by estrogen.

The pathophysiology underlying the relationship between sleep quality and youth ACS has been reported both in epidemiologic and experimental studies but is still not completely clear. It has been demonstrated that poor-quality sleep, as well as short-duration sleep or the presence of non-restorative sleep [39], can lead to sympathetic activation or increased levels of inflammatory cytokines in the plasma [41], both of which are associated with metabolic, endocrine, and cardiovascular diseases [43,44]. Meanwhile, evidence indicates that chronic hyperarousal and long-term stress responses caused by sleep disorders can motivate both the hypothalamic–pituitary–adrenal axis and the autonomic nervous system [45,46,47]; further increase the metabolic rate [48] and heart rate; and decrease heart rate variability [49]. These physiological processes may have an association with vascular endothelial dysfunction and atherosclerosis, which are responsible for the development of cardiovascular events in young ACS patients in later life.

Poor sleep quality may be one of the risk factors for young ACS patients and their disease’s adverse outcomes. Therefore, it is of great clinical significance to explore the association between sleep quality and coronary lesion complexity. The studies above indicate the importance of improving overall sleep quality for young ACS patients. However, the potential mechanism between sleep duration and youth ACS needs further exploration regarding the use of more drug-assisted therapy. Furthermore, health education about adjusting sleep habits and improving sleep should be carried out to promote public health in the future. A previous trial demonstrated that sleep quality can be improved by treating depressive symptoms in ACS patients [50], which could be applied to cardiac rehab programs for youth ACS patients.

This study has some limitations. First, the study was based on a single center, enrolling patients of East Asian heritage, and needs to be validated in diverse groups. Second, our sample size was relatively small, with a predominance of male patients, and we had a small number of outcome events with predominant TVR, which might affect the accuracy of the results to some extent. Third, compared with polysomnography, sleep quality questionnaires cannot provide objective data, and there could be a recall bias in self-reported PSQIs.

## 5. Conclusions

This study suggests that sleep quality is positively associated with coronary lesion complexity and that long-term poor sleep quality is a risk factor for complex coronary lesions. In addition, patients with poor sleep quality showed higher risks of MACEs and TVR compared with those with good sleep quality. Our findings could serve as a meaningful reference for preventing and rehabilitating ACS and its malignant outcomes in young people. More randomized controlled trials should be conducted in the future to further verify our conclusions.

## Figures and Tables

**Table 1:** The baseline clinical characteristics, laboratory parameters and angiographic findings of the patients according to PSQI score.

	All Patients (466)	PSQI ≤ 5 (202)	PSQI > 5 (264)	*p* Value
**Clinical Characteristics**				
Age (yrs.)	41 (37–43)	41 (37–43)	40 (37–43)	0.771
Men, *n* (%)	434 (93.13%)	188.0 (93.07%)	246 (93.18%)	0.962
BMI (kg/m^2^)	27.4 (25.28–29.4)	27.4 (25.48–29.37)	27.25 (25.02–29.45)	0.967
Hypertension, *n* (%)	263 (56.44%)	124 (61.39%)	139 (52.65%)	0.060
Diabetes mellitus, *n* (%)	140 (30.04%)	57 (28.22%)	83.0 (31.44%)	0.452
Hyperlipidemia, *n* (%)	413 (88.63%)	176 (87.13%)	237 (89.77%)	0.373
Smoker, *n* (%)	291 (62.45%)	128 (63.37%)	163 (61.74%)	0.720
**Diagnosis**				
UA, *n* (%)	326 (69.96%)	139 (68.81%)	187 (70.83%)	0.637
NSTEMI, *n* (%)	97 (20.82%)	45 (22.28%)	52 (19.7%)	0.497
STEMI, *n* (%)	43 (9.23%)	18 (8.91%)	25 (9.47%)	0.836
**Laboratory parameters**				
SBP (mmHg)	126.0 (118.0–135.0)	128.0 (118.0–137.0)	125.0 (118.0–134.0)	0.105
DBP (mmHg)	79.8 (70.0–88.0)	80.0 (71.0–89.75)	79.0 (70.0–86.0)	0.081
FBG (mmol/L)	5.78 (5.15–7.14)	5.88 (5.15–7.32)	5.74 (5.16–7.01)	0.296
HbA1C (%)	5.9 (5.5–6.23)	5.9 (5.5–6.23)	5.9 (5.5–6.23)	0.909
TG (mmol/L)	1.77 (1.32–2.27)	1.74 (1.19–2.29)	1.79 (1.36–2.27)	0.078
TC (mmol/L)	3.99 (3.4–4.78)	4.03 (3.34–4.81)	3.96 (3.41–4.71)	0.960
HDL-C (mmol/L)	0.96 (0.84–1.09)	0.95 (0.82–1.1)	0.97 (0.85–1.09)	0.445
LDL-C (mmol/L)	2.34 (1.84–2.97)	2.38 (1.88–3.14)	2.3 (1.82–2.82)	0.184
ALT (U/L)	31.5 (23.0–45.0)	33.0 (24.0–46.5)	31.0 (22.0–43.0)	0.185
AST (U/L)	25.0 (19.0–32.0)	25.0 (19.0–32.0)	24.0 (19.0–32.0)	0.330
Hcy (μmol/L)	11.95 (9.7–15.2)	11.7 (9.43–15.12)	12.3 (9.8–15.6)	0.205
Creatinine (μmol/L)	73.35 (65.45–81.0)	73.15 (66.0–80.5)	73.75 (65.1–81.22)	0.801
eGFR (mL/min/1.73 m^2^)	112.7 (106.96–118.43)	112.79 (107.3–117.77)	112.7 (106.52–118.55)	0.983
Uric acid (μmol/L)	396.16 ± 91.59	388.28 ± 88.26	401.55 ± 88.21	0.474
Hs-CRP (mg/L)	1.36 (0.58–3.3)	1.12 (0.5–3.26)	1.54 (0.72–3.38)	0.049
Tn I (ng/mL)	0.0 (0.0–0.2)	0.0 (0.0–0.34)	0.0 (0.0–0.1)	0.457
**Medicines Before Hospital**				
Aspirin, *n* (%)	297 (63.73%)	116 (57.43%)	181 (68.56%)	0.013
P2Y12 receptor inhibitor, *n* (%)	226 (48.5%)	95 (47.03%)	131 (49.62%)	0.579
β-receptor blocker, *n* (%)	73 (15.67%)	29 (14.36%)	44 (16.67%)	0.497
ACEI/ARB, *n* (%)	137 (29.4%)	57 (28.22%)	80 (30.3%)	0.624
CCB, *n* (%)	70 (15.02%)	35 (17.33%)	35 (13.26%)	0.223
Statin, *n* (%)	268 (57.51%)	105 (51.98%)	163 (61.98%)	0.031
**Angiographic Findings**				
SYNTAX Score	14 (7–22.5)	10 (7–18)	17 (8–24)	<0.001
SYNTAX Score ≤ 22, *n* (%)	348 (47.68%)	177 (87.62%)	171 (64.77%)	<0.001
SYNTAX Score >22, *n* (%)	118 (25.32%)	25 (12.38%)	93 (35.23%)	
Lesion branches				
One-vessel disease, *n* (%)	191 (40.99%)	96 (47.52%)	95 (35.98%)	0.012
Two-vessel disease, *n* (%)	141 (30.26%)	62 (30.69%)	79 (29.92%)	0.858
Three-vessel disease, *n* (%)	134 (28.76%)	44 (21.78%)	90 (34.09%)	0.004
Lesioned vessels				
LM, *n* (%)	32 (6.87%)	6 (2.97%)	26 (9.85%)	0.004
LAD, *n* (%)	385 (82.62%)	165 (81.68%)	220 (83.33%)	0.642
LCX, *n* (%)	234 (50.21%)	86 (42.57%)	148 (56.06%)	0.004
RCA, *n* (%)	252 (54.08%)	99 (49.01%)	153 (57.95%)	0.055
Lesion characteristics				
Total occlusions, *n* (%)	157 (33.69%)	51 (25.25%)	106 (40.15%)	0.001
Trifurcation/bifurcation lesions, *n* (%)	77 (16.52%)	21 (10.40%)	56 (21.21%)	0.002
Heavy calcification lesions, *n* (%)	31 (6.65%)	18 (8.91%)	13 (4.92%)	0.087
Thrombosis, *n* (%)	12 (2.585%)	5 (2.48%)	7 (2.65%)	0.905
Lesions > 20 mm long, *n* (%)	144 (30.90%)	62 (30.69%)	82 (31.06%)	0.932
Complete revascularization, *n* (%)	194 (41.63%)	75 (37.13%)	119 (45.08%)	0.085

Abbreviations: BMI = body mass index; UA = unstable angina; NSTEMI = non-ST-segment elevation myocardial infarction; STEMI = ST-segment elevation myocardial infarction; SBP = systolic blood pressure; DBP = diastolic blood pressure; FBG = fasting blood glucose; HbA1C = glycosylated hemoglobin A1C; TG = triglyceride; TC = total cholesterol; HDL-C = high-density lipoprotein cholesterol; LDL-C = low-density lipoprotein cholesterol; ALT = alanine transaminase; AST = aspartate transaminase; eGFR= estimated glomerular filtration rate; Hs-CRP = hyper-sensitive C-reactive protein; TnI = troponin I; ACEI = angiotensin-converting enzyme inhibitors; ARB = angiotensin receptor blocker; CCB = calcium channel blockers; LM = left main; LAD = left anterior descending; LCX = left circumflex; RCA = right coronary artery.

**Table 2:** Outcomes of the patients according to PSQI grades.

	All Patients (466)	PSQI ≤ 5 (202)	PSQI > 5 (264)	*p* Value
**Medicines After Hospital**				
Aspirin, *n* (%)	457 (98.07%)	196 (97.03%)	261 (98.86%)	0.154
Clopidogrel, *n* (%)	246 (52.79%)	104 (51.49%)	142 (53.79%)	0.622
Ticagrelor, *n* (%)	176 (37.77%)	72 (35.64%)	104 (39.39%)	0.408
Statin, *n* (%)	444 (95.28%)	196 (97.03%)	248 (93.94%)	0.119
β-receptor blocker, *n* (%)	332 (71.24%)	149 (73.76%)	183 (69.32%)	0.294
ACEI/ABR, *n* (%)	127 (27.25%)	62 (30.69%)	65 (24.62%)	0.145
CCB, *n* (%)	66 (14.16%)	36 (17.82%)	30 (11.36%)	0.048
**Outcomes**				
Follow up (ms.)	74 (69–77)	74 (69.25–76)	75 (63–77)	0.768
MACEs, *n* (%)	84 (18.03%)	12 (5.94%)	72 (27.27%)	<0.001
All-cause death, *n* (%)	4 (0.86%)	0 (0.0%)	4 (1.52%)	0.137
MI, *n* (%)	17 (3.65%)	5 (2.48%)	12 (4.55%)	0.238
TVR, *n* (%)	82 (17.60%)	12 (5.94%)	70 (26.52%)	<0.001
Cardiac death, *n* (%)	2 (0.43%)	0 (0.0%)	2 (0.76%)	0.508

Abbreviations: MACEs = include all-cause death, MI and TVR; MI = myocardial infarction; TVR = target-vessel revascularization.

**Table 3:** Logistic regression analysis of association between sleep characteristics and coronary lesion complexity.

	Model 1		Model 2		Model 3	
	OR (95%CI)	*p* Value	OR (95%CI)	*p* Value	OR (95%CI)	*p* Value
Time for bed	1.410 (1.133, 1.756)	0.002	1.468 (1.170, 1.842)	0.001	1.457 (1.156, 1.836)	0.001
Sleep duration	0.763 (0.629, 0.926)	0.006	0.760 (0.624, 0.926)	0.007	0.775 (0.633, 0.948)	0.013
PSQI score	1.268 (1.173, 1.371)	<0.001	1.259 (1.163, 1.363)	<0.001	1.264 (1.166, 1.371)	<0.001
PSQI grade	3.852 (2.362, 6.282)	<0.001	3.748 (2.282, 6.154)	<0.001	3.846 (2.313, 6.394)	0.001

Model 1: adjusted for sex and age. Model 2: adjusted for sex, age, BMI, hypertension, diabetes mellitus, hyperlipidemia, and smoker. Model 3: adjusted for sex, age, BMI, hypertension, diabetes mellitus, hyperlipidemia, smoker, FBG, HbA1c, HDL-C, Uric acid, and Hcy.

**Table 4:** Cox proportional hazards regression of PSQI grade and clinical outcomes.

Variable	All Patients (466)	PSQI ≤ 5 (202)	PSQI > 5 (264)	HR (95%*CI*)	*p* Value
Follow up (ms.)	74 (69, 77)	74 (69.25, 76)	75 (63, 77)		
MACEs, *n* (%)	84 (18.03%)	12 (5.94%)	72 (27.27%)	4.266 (2.274, 8.001)	<0.001
All-cause death, *n* (%)	4 (0.86%)	-	4 (1.52%)	-	0.812
MI, *n* (%)	17 (3.65%)	5 (2.48%)	12 (4.55%)	1.176 (0.382, 3.624)	0.778
TVR, *n* (%)	82 (17.60%)	12 (5.94%)	70 (26.52%)	4.073 (2.169, 7.652)	<0.001
Cardiac death, *n* (%)	2 (0.43%)	-	2 (0.76%)	-	0.789

Adjusted for sex, age, hypertension, diabetes mellitus, hyperlipidemia, smoker, HbA1c, LDL-C, hs-CRP, complete revascularization, and SYNTAX score.

## Data Availability

The data are not publicly available due to their containing information that could compromise the privacy of research participants.

## Supplementary Materials

The following supporting information can be downloaded at https://www.mdpi.com/article/10.3390/jcdd11020068/s1, Supplementary Table S1. Univariate Logistic regression analysis between covariates and SYNTAX grades. Supplementary Table S2. The collinearity check between covariates in model 3 of logistic regression analysis. Supplementary Table S3. Univariate Cox proportional hazards regression analysis of covariates and clinical outcomes. Supplementary Table S4. The collinearity check between covariates in adjusted model of Cox proportional hazards regression analysis.
