# Supplementary material for: The Effect of Sleep Quality on Coronary Lesion Severity and Prognosis in the Young Acute Coronary Syndrome Population
Source: J Cardiovasc Dev Dis. 2024 Feb 19;11(2):68. doi: 10.3390/jcdd11020068 (PMC10889764; doi:10.3390/jcdd11020068)
Supplement: Supplementary file 1 [file jcdd-11-00068-s001.zip › jcdd-2850179-supplementary.pdf]

Supplementary Table S1. Univariate Logistic regression analysis between covariates and SYNTAX group.

	OR (95%CI)	*P* value
Age	0.995(0.94,1.053)	0.860
Men	1.227(0.517,2.916)	0.643
Body mass index	1.047(0.983,1.116)	0.156
Hypertension	0.773(0.509,1.176)	0.230
Diabetes mellitus	1.726(1.113,2.678)	0.015
Hyperlipidemia	2.042(0.933,4.468)	0.074
Smoker	0.727(0.475,1.113)	0.142
Systolic blood pressure	1.003(0.990,1.016)	0.635
Diastolic blood pressure	0.993(0.977,1.010)	0.416
Fasting blood glucose	1.067(1.004,1.135)	0.038
HbA1c (%)	1.151(0.988,1.342)	0.071
Triglyceride	1.064(0.921,1.229)	0.400
Total cholesterol	1.019(0.847,1.225)	0.843
High density lipoprotein cholesterol	0.241(0.077,0.755)	0.015
Low density lipoprotein cholesterol	1.052(0.849,1.302)	0.645
Alanine transaminase	0.996(0.989,1.004)	0.345
Aspartate transaminase	0.998(0.993,1.003)	0.440
Hcy (umol/L)	1.017(0.997,1.037)	0.101
Creatinine (umol/L)	1.007(0.993,1.021)	0.337
eGFR (mL/min)	0.992(0.975,1.009)	0.337
Uric acid (umol/L)	1.002(1.000,1.005)	0.053
Hyper-sensitive C-reactive protein	1.022(0.982,1.063)	0.293
Troponin I	0.986(0.955,1.017)	0.366
Time for bed	1.413(1.136,1.758)	0.002
Sleep duration	0.764(0.630,0.927)	0.006
PSQI score	1.265(1.171,1.368)	<0.001

PSQI grade	3.851(2.361,6.279)	<0.001

HbA1C=glycosylated hemoglobin A1C; PSQI=Pittsburgh Sleep Quality Index; PSQI score: a continuous variable; PSQI grade: a dichotomous variable; OR, odds ratio; CI, confidence interval.

Supplementary Table S2. The collinearity check between covariates in model 3 of logistic regression analysis.

Model	Unstandardized Coefficients		Standardized Coefficients	t	Sig.	Collinearity Statistics	
	B	Std. Error	Beta			Tolerance	VIF
(Constant)	-0.511	0.3		-1.703	0.089		
Age	-0.049	0.076	-0.033	-0.652	0.515	0.784	1.276
Men	0.003	0.005	0.025	0.557	0.578	0.958	1.044
BMI	0.004	0.006	0.037	0.769	0.442	0.841	1.19
Hypertension	0.002	0.035	0.002	0.054	0.957	0.943	1.061
Diabetes mellitus	-0.076	0.052	-0.091	-1.465	0.144	0.512	1.953
Hyperlipidemia	0.093	0.054	0.077	1.717	0.087	0.968	1.033
Smoker	-0.056	0.037	-0.071	-1.492	0.136	0.88	1.136
FBG	0.007	0.006	0.056	1.182	0.238	0.891	1.122
HbA1c	0.035	0.018	0.116	1.962	0.05	0.564	1.773
HDL-C	0.008	0.094	0.004	0.083	0.934	0.873	1.145
Uric acid	0	0	0.047	0.978	0.328	0.853	1.172
Hcy	0.001	0.002	0.017	0.365	0.715	0.937	1.067
PSQI grade	0.215	0.035	0.278	6.183	0	0.974	1.027

Dependent Variable: SYNTAX score

BMI=body mass index; FBG=fasting blood glucose; HbA1C=glycosylated hemoglobin A1C; HDL-C=high density lipoprotein cholesterol; PSQI=Pittsburgh Sleep Quality Index; PSQI grade: a dichotomous variable.

Supplementary Table S3. Univariate Cox proportional hazards regression analysis of covariates and Clinical outcomes

	HR (95%CI)	*P* value
Age	1.011(0.953,1.072)	0.721
Men	0.748(0.345,1.622)	0.462
Body mass index	1.038(0.976,1.105)	0.236
Hypertension	0.945(0.613,1.456)	0.796
Diabetes mellitus	1.060(0.667,1.686)	0.804
Hyperlipidemia	2.687(0.984,7.337)	0.054
Smoker	0.692(0.449,1.066)	0.095
Systolic blood pressure	1.006(0.992,1.019)	0.405
Diastolic blood pressure	1.002(0.985,1.02)	0.785
Fasting blood glucose	1.024(0.97,1.081)	0.391
HbA1c (%)	1.114(0.963,1.289)	0.145
Triglyceride	1.084(0.952,1.234)	0.223
Total cholesterol	0.864(0.703,1.062)	0.165
High density lipoprotein cholesterol	1.057(0.353,3.159)	0.921
Low density lipoprotein cholesterol	0.789(0.614,1.015)	0.066
Alanine transaminase	0.989(0.978,1)	0.245
Aspartate transaminase	0.998(0.992,1.004)	0.466
Hcy (umol/L)	1.003(0.983,1.024)	0.755
Creatinine (umol/L)	1.005(0.99,1.02)	0.487
eGFR (mL/min)	0.99(0.973,1.007)	0.229
Uric acid (umol/L)	1.002(0.999,1.004)	0.183
Hyper-sensitive C-reactive protein	1.04(1.005,1.077)	0.027
Troponin I	1(0.978,1.023)	0.99
Complete revascularization	0.648(0.412,1.020)	0.061
SYNTAX score	1.048(1.027,1.068)	<0.001
PSQI grade	5.468(2.897,10.319)	<0.001

HbA1C=glycosylated hemoglobin A1C; PSQI=Pittsburgh Sleep Quality Index; PSQI grade: a dichotomous variable; HR, hazard ratio; CI, confidence interval.

Supplementary Table S4. The collinearity check between covariates in adjusted model of Cox proportional hazards regression analysis

Model	Unstandardized Coefficients		Standardized Coefficients			Collinearity Statistics	
	B	Std. Error	Beta	t	Sig.	Tolerance	VIF
(Constant)	-0.14	0.227		-0.616	0.538		
Age	-0.042	0.07	-0.028	-0.595	0.552	0.886	1.129
Men	0.001	0.005	0.007	0.154	0.878	0.985	1.015
Hypertension	0.007	0.034	0.009	0.199	0.842	0.982	1.018
Diabetes mellitus	-0.078	0.048	-0.094	-1.627	0.104	0.571	1.751
Hyperlipidemia	0.098	0.053	0.081	1.851	0.065	0.977	1.024
Smoker	-0.058	0.037	-0.074	-1.597	0.111	0.885	1.131
HbA1c	0.029	0.017	0.096	1.669	0.096	0.568	1.759
LDL-C	-0.043	0.018	-0.107	-2.392	0.017	0.939	1.065
Hs-CRP	0.008	0.003	0.107	2.36	0.019	0.924	1.082
Complete revascularization	-0.076	0.034	-0.098	2.238	0.026	0.982	1.019
SYNTAX Score	0.006	0.002	0.16	3.486	0.001	0.902	1.109
PSQI grade	0.179	0.035	0.232	5.106	0	0.914	1.094

Dependent Variable: MACEs

HbA1C=glycosylated hemoglobin A1C; LDL-C=low density lipoprotein cholesterol; Hs-CRP=hyper-sensitive C-reactive protein; PSQI=Pittsburgh Sleep Quality Index; PSQI grade: a dichotomous variable.
